# Emergency room visits for respiratory conditions in children increased after Guagua Pichincha volcanic eruptions in April 2000 in Quito, Ecuador Observational Study: Time Series Analysis

**DOI:** 10.1186/1476-069X-6-21

**Published:** 2007-07-24

**Authors:** Elena N Naumova, Hugo Yepes, Jeffrey K Griffiths, Fernando Sempértegui, Gauri Khurana, Jyotsna S Jagai, Edgar Játiva, Bertha Estrella

**Affiliations:** 1 Department of Public Health and Family Medicine, Tufts University School of Medicine, Boston MA 02111, USA; 2 Instituto Geofisico, Escuela Politecnica Nacional, Quito, Ecuador; 3 Corporación Ecuatoriana de Biotecnología, Quito, Ecuador; 4 Baca Ortiz Children's Hospital, Quito, Ecuador

## Abstract

**Background:**

This study documented elevated rates of emergency room (ER) visits for acute upper and lower respiratory infections and asthma-related conditions in the children of Quito, Ecuador associated with the eruption of Guagua Pichincha in April of 2000.

**Methods:**

We abstracted 5169 (43% females) ER records with primary respiratory conditions treated from January 1 – December 27, 2000 and examined the change in pediatric ER visits for respiratory conditions before, during, and after exposure events of April, 2000. We applied a Poisson regression model adapted to time series of cases for three non-overlapping disease categories: acute upper respiratory infection (AURI), acute lower respiratory infection (ALRI), and asthma-related conditions in boys and girls for three age groups: 0–4, 5–9, and 10–15 years.

**Results:**

At the main pediatric medical facility, the Baca Ortiz Pediatric Hospital, the rate of emergency room (ER) visits due to respiratory conditions substantially increased in the three weeks after eruption (RR = 2.22, 95%CI = [1.95, 2.52] and RR = 1.72 95%CI = [1.49, 1.97] for lower and upper respiratory tract infections respectively. The largest impact of eruptions on respiratory distress was observed in children younger than 5 years (RR = 2.21, 95%CI = [1.79, 2.73] and RR = 2.16 95%CI = [1.67, 2.76] in boys and girls respectively). The rate of asthma and asthma-related diagnosis doubled during the period of volcano fumarolic activity (RR = 1.97, 95%CI = [1.19, 3.24]). Overall, 28 days of volcanic activity and ash releases resulted in 345 (95%CI = [241, 460]) additional ER visits due to respiratory conditions.

**Conclusion:**

The study has demonstrated strong relationship between ash exposure and respiratory effects in children.

## Background

The danger of living near active volcanoes has been well documented since ancient times, from the ruins of Pompeii to the recent satellite images of volcano eruptions. Volcanic activity is typically associated with toxic emissions; pyroclastic flows (the mixture of rock fragments and superheated gases, which can achieve speeds over 100 km/hr and temperatures over 300°C); the release of ash; volcanic gases containing carbon dioxide, water vapor, sulfur dioxide, hydrogen chloride, hydrogen sulphide, hydrogen fluoride, etc.; and polycyclic aromatic hydrocarbons produced whenever any complex organic material is burned by hot pyroclastic flows [1,2]. Although approximately 455 million people worldwide live within potential exposure range of an active volcano, information on the acute and chronic respiratory health effects of volcanic emissions is sparse [3,4].

The 1980 eruption of Mt. St. Helens [5] led to numerous studies of health effects of volcanic emissions. The acute effects of volcanic ash falls and gases on respiratory conditions vary from undetected to well-defined [2,3]. Transient acute irritant effects of volcanic ash and gases on the mucous membranes of upper respiratory tract and exacerbation of chronic lung diseases during and shortly after eruptions with heavy ash fall have been documented [6,7]. Such relationships have been found following the more recent eruption in Cerro Negro, Nicaragua in 1992 [8], Mt. Sakura-jima in Japan [9], and Mt. Tungurahua in Ecuador [10].

**Figure 1:**
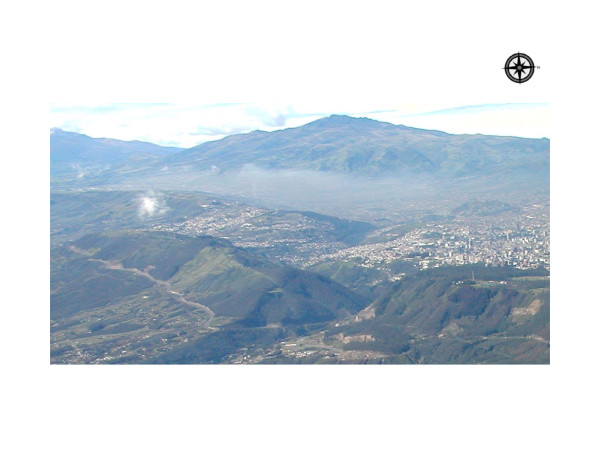
Aerial photograph of Quito, the capital of Ecuador. Footnotes: The Pichincha volcano located west of Quito is in the central background of the picture. Along its base is the narrow strip with an air pollution haze stretching from left to right (south to north) across the city.

Quito, the capital of Ecuador, located 2800 m above sea level, is surrounded by four active volcanoes: Guagua Pichincha, Cotopaxi, Antisana, and Tungurahua [11]. The stratovolcano Guagua Pichincha is located 13 km (7 mi) west of Quito and became active in 1998 after 340 years of dormancy with emissions of vapor, ashes, and fumes. In the spring of 2000, volcanic ashes containing silica, sulfurs, and particulate matter were again reported and yellow alerts were issued for the city of Quito. Nestled in a long, narrow valley between the base of the mountain range to the west and the precipitous canyon of the Machángara River to the east, Quito possesses an unmatched setting: a dangerous chamber created by the nature and enhanced by humans (Figure 1). Over the last two decades, the rapid urbanization and sprawl has resulted in hazardously high levels of air pollutants [12,13]. Quito's unique geographical location, with its topology and the pattern of prevailing wind, traps stagnant contaminated air. At that altitude, where the demand for oxygen is high, even a small increase in air pollution may trigger a substantial increase in ER visits for respiratory conditions. In fact, our own studies indicate strong effects of air pollution on respiratory health in Quito's children [14].

Recent work has emphasized a complex spectrum of health problems associated with direct and indirect effects of displacement, disruptions in infrastructure [15,16], soil, air, and water contamination [17,18]. Studies stress the need for implementing efficient surveillance systems to monitor the health effects associated with various environmental and socio-economic factors, especially in the most vulnerable populations [8]. In the absence of such surveillance systems, we have made an attempt to characterize the respiratory health outcomes that often reflect the most severe conditions requiring urgent ER visits and hospitalization. In the presented study we describe the change in pediatric ER visits for respiratory conditions before, during, and after ash falls in Quito due to Pichincha volcano eruptions which occurred in April, 2000. We believe that the results of this study provide important information for environmental public health policy and better understanding of the short term effects of environmental exposure to volcanic ashes on human health.

## Data and Methods

### Study population and outcomes

This study was conducted at the Baca Ortiz Children's Hospital, Quito's largest government-subsidized facility, which provides care to approximately 307,000 children under 15 years of age residing in the Quito Metropolitan area and surrounding communities [19]. During 2000, 72,039 children were treated at the hospital's outpatient departments, 22,353 children were assisted at the hospital's emergency room, and about six per cent of children visited ER were treated for respiratory illnesses, mainly pneumonia (Department of Statistics, Baca Ortiz Hospital, 2000, unpublished data). The Ethical Committee of Ecuador and the Tufts IRB granted permission for abstraction of de-identified records.

We abstracted 5169 (43% females) ER records with primary respiratory conditions treated from January 1 – December 27, 2000. For each case the following information was recorded: age, sex, residential location (barrios), admission date, temperature, weight, respiratory rate, and primary diagnosis. For 4416 (85%) children nutritional status was determined using EPI/Info software [20] and classified as underweight if the weight-for-age z-score was more than two standard deviations below the age adjusted mean and normal otherwise. Based on the primary diagnosis given in the ER and similar to previous study [14] we classified records into three non-overlapping categories: acute upper respiratory infection (AURI), which includes conditions coded as pharyngitis, tonsillitis, sinusitis, rhinitis, laryngitis, and their combinations; acute lower respiratory infection (ALRI), which includes conditions coded as pneumonia, bronchopneumonia, traqueobronchitis, bronchitis; and asthma-related conditions (2392, 2319, and 431 cases in each group respectively). We estimated the annual incidence rate for ER visits (per 1000 children) in boys and girls for three age groups: 0–4, 5–9, and 10–15 years using the estimates of population served calculated from census data [18].

### The timing of eruption

**Figure 2:**
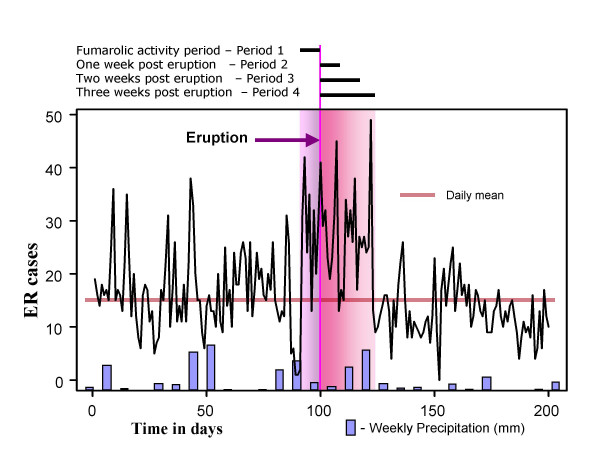
The time line of the Guagua Pichincha volcano activity, daily ER visits, and weekly precipitation. Footnotes: Time series of daily cases of ER visits for first 200 days are shown. The most noticeable fumarolic activity was observed a week prior eruptions (shown as a pink bar). On April 10^th ^and 11^th ^(days 100 and 101), the volcano erupted approximately 18 times. More than 100 seismic tremors were recorded during the eruptions. Ash clouds rose 0.8 km into the air on April, 10^th ^and then another cloud of vapor was seen on April 16^th^. A yellow alert was issued for the city Quito. The study periods (2, 3, and 4) are indicated as a red bar.

We abstracted information on Guagua Pichincha activity from various sources, including Instituto Geofisico de la Escuela Politecnica Nacional, Volcano Ash Advisory Archive, maintained by the NOAA Satellite and Information Service [21], and the main local news media, and created a timeline of exposure-related volcanic activity. Figure 2 illustrates the compiled timeline superimposed on the time series of daily counts of respiratory infections in the studied population. In the spring of 2000 Pichincha was active; in February, plumes rose to 1–2 km often carrying noticeable ash, and a few ash falls were seen near the vent. During January to April, fumarolic activity was moderate but variable, often most noticeable a week before eruptions (shown as a pink bar in Figure 2). Between April 9 and12 (days 99–102 in the Figure 2), the volcano erupted approximately 18 times. Ash clouds rose 0.8 km into the air. More than 100 seismic tremors were recorded during the eruptions. A yellow alert was issued for the city on April 12. On April 16, the volcano sent clouds of vapor 0.5 km into the sky over Quito. We defined four periods for analysis: fumarolic activity period – Period 1, one week post eruption – Period 2, two weeks post eruption – Period 3, and three weeks post eruption – Period 4. We also abstracted daily measures for temperature and precipitation, obtained from the Instituto Nacional de Meteorología e Hidrología of Ecuador [22].

### Models and analysis

Six time series of daily counts of ER visits were compiled based on the date of admission: for males and females in each of the three age categories. In 52 (1%) records, a missing day was replaced with a mid-month value. Pair-wise Spearman correlations between time series for boys and girls ranged from 0.4 to 0.7 (all significant) indicating similarity of temporal patterns. Three time series of daily counts of ER visits for each diagnostic group for all children were created.

We also compiled a set of binary time series to indicate the timing of volcano eruptions including one, two, and three week post-eruption periods (shown as red bar in Figure 2) and one week prior eruption (shown as pink bar in Figure 2). To examine the relationship between the timing of the volcanic eruption and ER visits we applied a Poisson regression model adapted to time series data adjusted for effects of the day of the week, official holidays and potential effect of replaced missing dates, and meteorological factors: log [Y(t)] = β_0_+β_1_E+β_2_D+β_3_H+β_4_P+ε(t), where Y(t) is a time series of cases, and βs are regression parameter for the variables reflecting presence of exposure (E), weekly cycle (D), holidays and mid-month indicator (H), and weekly precipitation level (P). The predicted daily rate of ER visits per 1000 children; the relative risks (RR), and their 95% confidence intervals (95%CI) were estimated for boys and girls in three age groups for all ER visits and for three diagnostic groups in all children. To examine if the higher rate of ER visits in malnourished children was associated with exposure we performed the χ^2^-test.

To characterize the spatial distribution of ER cases we geocoded 4786 (93.4% of all records) and categorized patients' residential locations into three regions: the City of Quito, the city suburbs, and the area outside the city metropolitan boundaries. Locations of Quito residents were further subdivided into sixteen parishes and five large suburban areas. The spatial distribution of cases and its change during the eruption period were examined.

## Results

The descriptive characteristics of population served by the Baca Ortiz Hospital and annual incidence rates of ER visits due to respiratory infections are shown in Table 1. The mean age for girls and boys was 3.23 ± 3.10 years and 3.18 ± 3.08 years, respectively. The annual rates of infections were consistently higher in males in every age category.

**Table 1:** Annual incidence rates of ER visits for three specific diagnostic groups.

		**Age groups**			
		**0 – 4**	**5 – 9**	**10 – 15**	**all ages**

Population served^a^					
	females	49860	50964	61267	162091
	males	51332	52160	61709	165201
Annual rate^b^					
	females	32.83^c^	8.37	2.57	13.70
	males	42.43	11.38	2.88	17.85
Annual rate of AURI					
	females	16.02	3.21	0.92	6.28
	males	21.39	4.37	0.78	8.32
Annual rate of ALRI					
	females	15.53	3.36	1.00	6.21
	males	19.47	4.72	1.19	7.98
Annual rate of Asthma					
	females	1.11	1.78	0.64	1.14
	males	1.43	2.24	0.90	1.49

^a ^Values are estimated from the Census 2000, Ecuador. ^b ^Annual rates are estimated per 1000 children served by the Baca Ortiz Hospital. ^c^Estimated as: (number of cases/population served) × 1000

Based on the results of the regression model we estimated the daily means of ER visits for a period that include time before and three weeks after the eruption (344 days). We also estimated the relative risks of the increase in the daily mean ER visits during the four defined periods of volcanic activity (28 days). These estimates are shown in Table 2. Overall, children under 4 years of age were treated at the ER three times more often then older children. At baseline, the daily mean ER visits was significantly higher in the youngest children compared to children older than 5 y.o. (0.092; 95%CI = [0.088, 0.098] vs. 0.024; 95%CI = [0.021, 0.027], p < 0.0001). The daily means of ER visits in the youngest children were two times higher during all four defined periods of volcanic activity than pre and post eruption after adjusting for the baseline differences. The relative risks for ER visitations were similar in boys and girls (e.g. during one-week post eruption: RR = 2.21, 95%CI = [1.79; 2.73] and RR = 2.16, 95%CI = [1.69; 2.76], respectively). These findings indicate that increases in daily cases relative to the annual average (e.g. Figure 2 – red horizontal line) were significant for all ER visits and each disease group.

**Table 2:** Predicted daily rates of ER visits and the relative risks associated with the volcanic activity

	**Age groups**	Males			**Females**		
		
		**estimate**	**LCI**	**UCI**	**estimate**	**LCI**	**UCI**
Daily rates^a^							
	0–4	0.104	0.099	0.110	0.080	0.076	0.086
	5–9	0.027	0.024	0.030	0.020	0.018	0.023
	10–15	0.007	0.006	0.009	0.007	0.005	0.008
	all	0.043	0.041	0.045	0.040	0.039	0.042
Relative Risks^b^							
Fumarolic activity period – Period 1							
	0–4	**2.008**	1.610	2.504	**1.953**	1.509	2.527
	5–9	**1.698**	1.075	2.681	1.358	0.746	2.472
	10–15	1.180	0.438	3.180	1.681	0.690	4.098
	all	**1.927**	1.587	2.339	**1.799**	1.458	2.220
One week post eruption – Period 2							
	0–4	**2.212**	1.791	2.732	**2.156**	1.685	2.758
	5–9	1.331	0.797	2.222	1.106	0.571	2.140
	10–15	2.102	0.987	4.476	0.996	0.318	3.121
	all	**1.965**	1.621	2.381	**1.799**	1.458	2.220
Two weeks post eruption – Period 3							
	0–4	**1.995**	1.699	2.344	**2.280**	1.913	2.718
	5–9	**1.434**	1.004	2.048	1.108	0.691	1.777
	10–15	**1.820**	1.013	3.270	0.488	0.156	1.530
	all	**1.795**	1.552	2.076	**1.894**	1.632	2.199
Three weeks post eruption – Period 4							
	0–4	**2.056**	1.799	2.350	**2.096**	1.798	2.442
	5–9	**1.579**	1.188	2.100	1.067	0.717	1.586
	10–15	1.625	0.972	2.716	0.879	0.431	1.790
	all	**1.891**	1.678	2.130	**1.807**	1.591	2.052

^a^estimated baseline together with 95% confidence interval (LCI, UCI) for cases per day per 1000 served population before and after volcanic eruption.

^b ^relative risks together with 95% confidence interval (LCI, UCI) for cases per day per 1000 served population for effects of the day of the week, official holidays and weekly precipitation.

On average, the predicted rates of ER visits for acute upper and lower respiratory tract infections were significantly higher (from 1.65 to 2.32 times) during all four predetermined periods compared to the rates in pre and post eruption periods (Table 3). The rate of asthma and asthma-related diagnosis was two times higher during the period of fumarolic activity RR = 1.97, 95%CI = [1.19, 3.24], but not during other time periods. Eight percent of children admitted to ER were underweight. No difference in the rates of ER visits between underweight and normal children associated with exposure was observed (χ^2^-test; p = 0.24).

**Table 3:** The relative risks associated with the volcanic activity for three diagnostic groups.

	**ALRI (n = 2392)**			**AURI (n = 2319)**			**ASTHMA (n = 431)**		
	**RR^a^**	**LCI**	**UCI**	**RR**	**LCI**	**UCI**	**RR**	**LCI**	**UCI**

Period 1^b^	**2.056**	1.665	2.538	**1.654**	1.314	2.082	**1.966**	1.193	3.239
Period 2^c^	**2.318**	1.899	2.830	**1.677**	1.334	2.107	1.586	0.913	2.754
Period 3^d^	**2.125**	1.827	2.473	**1.808**	1.541	2.122	1.089	0.679	1.746
Period 4^e^	**2.221**	1.959	2.518	**1.716**	1.497	1.968	0.963	0.638	1.453

^a^The relative risks and their 95% confidence intervals (LCI, UCI for all ages combined) of an increase in the daily mean ER visits during the defined periods for three diagnostic groups.

^b^Fumarolic activity period – Period 1

^c^One week post eruption – Period 2

^d^Two weeks post eruption – Period 3

^e^Three weeks post eruption – Period 4

**Figure 3:**
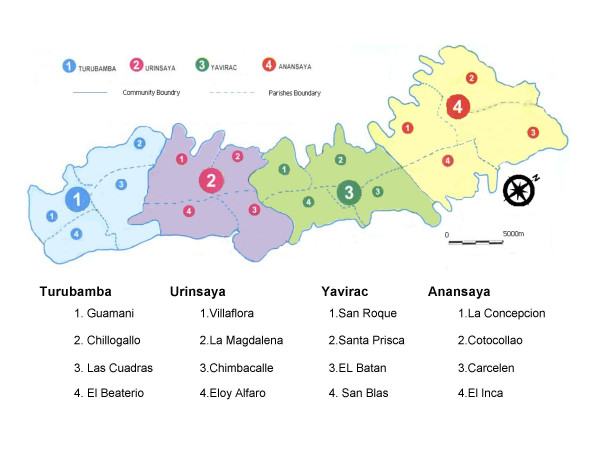
A schematic map of the city of Quito. Footnotes: Names of parishes from where geocoded residential locations of patients that visited the emergency room of the Baca Ortiz Hospital in 2000 correspond to Table 4.

An observed 2.25-fold increase in daily ER visits was present in all parts of the city (Table 4 and Figure 3). The suburb residents accounting for 20% of ER visits experienced a 3-fold increase (3.05) in daily counts of ER visits during the volcano eruption period. Overall, during 28 days of volcanic activity and ash releases on average 138 girls 95%CI = [104, 207], and 206 boys 95%CI = [137, 252], were treated in ER in addition to typically observed level of 6.17 and 8.18 cases per day in girls and boys respectively.

**Table 4:** Geographical distribution of residential locations of patients visited emergency room visits at two periods.

		**Pre and post eruption (334 days)**		**During eruption (28 days)**			
				
**Region**	**Parishes**	**Total cases**	**Cases per day**	**Total cases**	**Cases per day**	**Ratio**	**Percentage**
**City of Quito **(n = 405,206)^a^							
Turubamba (South)		**443**	**1.33**	**85**	**3.04**	**2.29**	**11**
	Guamani	64	0.19	8	0.29	1.49	2
	Chillogallo	219	0.66	43	1.54	2.34	5
	Las Cuadras	120	0.36	26	0.93	2.58	3
	El Beaterio	40	0.12	8	0.29	2.39	1
Urinsaya (South Central)		**902**	**2.70**	**173**	**6.18**	**2.29**	**22**
	Villaflora	333	1.00	69	2.46	2.47	8
	La Magdalena	140	0.42	25	0.89	2.13	3
	Chimbacalle	141	0.42	24	0.86	2.03	3
	Eloy Alfaro	288	0.86	55	1.96	2.28	7
Yavirac (North Central)		**1095**	**3.28**	**234**	**8.36**	**2.55**	**28**
	San Roque	321	0.96	64	2.29	2.38	8
	Santa Prisca	180	0.54	36	1.29	2.39	5
	EL Batan	66	0.20	14	0.50	2.53	2
	San Blas	528	1.58	120	4.29	2.71	14
Anansaya (North)		**936**	**2.80**	**162**	**5.79**	**2.06**	**23**
	La Concepcion	168	0.50	24	0.86	1.70	4
	Cotocollao	201	0.60	43	1.54	2.55	5
	Carcelen	333	1.00	53	1.89	1.90	8
	El Inca	234	0.70	42	1.50	2.14	6
**Quito suburbs **(n = 136,466)^a^		**422**	**1.26**	**108**	**3.86**	**3.05**	**11**
	Valle Tumabco (East)	79	0.24	24	0.86	3.62	2
	San Antonio (North)	48	0.14	11	0.39	2.73	1
	Calderon (North East)	159	0.48	41	1.46	3.08	4
	Valle de los Chillos (South East)	136	0.41	32	1.14	2.81	4
	Amaguana (South)	10	0.03	2	0.07	2.39	0.3
**Outside of Quito Metropolitan Area**		**169**	**0.51**	**45**	**1.61**	**3.18**	**4**
	**Total geocoded cases**	**3977**	**11.91**	**809**	28.89	2.43	100

^a^the numbers in parenthesis represent the population of children under 15 years of age.

## Discussion

This study documented elevated rates of ER visits for acute upper and lower respiratory infections and asthma-related conditions in the pediatric population of Quito associated with the eruption of Guagua Pichincha in April of 2000. Although Pichincha activity during the year 2000 was generally more moderate than in 1999, the rate of pediatric ER visits due to respiratory conditions following volcanic eruptions in April of 2000 doubled. The rate of asthma and asthma-related diagnosis was two times higher during the period of fumarolic activity of Pichincha. The largest increase in ER visitations was observed in the youngest children.

We demonstrated the potential of ER record utilization for monitoring the health effects associated with volcanic activity. Our study illustrates a set of analytical tools useful as the basis for pre-disaster planning and preparedness in areas where explosive and effusive volcanic eruptions are relatively common in proximity to vulnerable populations. Observed associations imply a potential scenario for compositions of immediate and delayed health effects following single or multiple exposure releases [23]. The onset of respiratory symptoms can manifest as three distinct waves. The first wave is composed of symptoms associated with the transient acute irritant effects of volcanic ash and gases on the mucous membranes of the eyes and upper respiratory tract, evidenced by the increase in asthma-related diagnosis which doubled during the period of fumarolic activity. The second and the third waves reflect conditions apparent with a specific delay and include acute upper and lower respiratory infections.

Volcanic ash is capable of inducing acute respiratory problems in susceptible people especially children and those with history of respiratory illness such as asthma or bronchitis [6,3,24,25]. The precise mechanism of those respiratory problems has not been well defined; however, its complexity has been outlined [3,26]. The basic etiology of lung inflammation begins with deposition of the particles into the lung. The location of the particle's deposition can be influenced by breathing pattern, the branched morphology of the airways, particle size and shape [1]. If a particle lodges in the main airways, it is cleared by the mucociliary escalator [27]. If it penetrates into the non-ciliated alveolar level, it may be cleared by the phagocytic alveolar macrophages. However, in the latter scenario, the particles may impair macrophage-mediated removal and these particles are not cleared. Some particles may directly penetrate the alveolar epithelium and reach the lung's deep lymph node drainage. Macrophages have also been shown to become inundated with inert particles, which subsequently halt the clearance of all particles at the alveolar level [28]. Macrophages release toxins which are utilized to destroy particles; however the released IL-1, IL-6, tumor necrosis factor (TNF), fibroblast growth factor, and the affluence of polymorphonuclear neutrophils damage the host's lung tissue [1]. The short-term effect of the above processes is lung inflammation, which manifests itself as difficulty in breathing and increased susceptibility to respiratory infections. Long-term prognoses include possibly fibrosis and carcinogenesis [29].

The irritant effect of volcanic ash on human airways depends on the physical and chemical proprieties of the ash including particulate size [6,30], the concentration of respirable ash particles, mineralogical composition, and duration of exposure [3]. It also depends on main features of the respiratory tract such as ventilation rate, the nasal filtration efficiency, and the mucocilliary clearance rate [30]. Several *in vitro *and *in vivo *experiments on different lung cells and animals suggest that inhaled volcanic ash is less toxic to the lung than other compounds like fine and coarse ambient particulate matter, quartz, or free crystalline silica. Some observations support the notion that volcanic ash is not a potent stimulus to lung inflammation since it does not stimulate the release of IL-8, a quimiotactic factor for neutrophils, nor does it depress γ-interferon and TNF-α secretion from either human alveolar macrophages or normal human bronchial epithelial cells [31,32]. However, it has been shown that physical immunologic barriers such as cilliary beating frequency and mucous lining can be altered after a short exposure to volcanic ash [33]. It has been reported that humoral immunologic parameters can also be affected by volcanic ash; for instance, workers exposed to volcanic ash had significantly lower C3 and C4 levels as well as marked decrease in serum IgG levels when compared to unexposed controls [34]. Immunological assays have shown that IgA, IgG, and albumin, airways proteins, play a protective role in mediating the effect of inhaled dust in human lungs [30]. Therefore, we hypothesize that young and malnourished children with pre-existing low levels of immunoglobulins, could be at higher risk for respiratory problems than healthy children. We did not find a difference in respiratory distress between underweight and normal children, potentially due to low sensitivity of weight-for-age measures in older children, but the age difference was well pronounced.

Moreover, some rodent models suggest that exposure to different particulate air pollutants including volcanic ash, stimulates immunocompetent cells, e.g. monocytes, more strongly than alveolar macrophages, to produce oxidative response [35]. Exposure to volcanic ash was also found to be related to an impairment of stimulated superoxide production while resting superoxide anion production is normal suggesting that volcanic ash might pose a risk for infection by compromising phagocyte antibacterial functions [36]. Such mechanisms may explain why individuals with underlying lung impairment including chronic inflammation are more susceptible to harmful effect of air pollution than healthy individuals. At Quito's altitude, where the demand for oxygen is high, even a small increase in CO, SO_2_, CO_2_, and similar compounds that might affect hematocrit level (and are already known to be associated with asthma exacerbation) may trigger a substantial increase in ER visits for asthma exacerbation especially during volcano's fumarolic activity.

Very little is known on the effects of "fresh" fractured silica particles on the developing lung tissue in young children. One may speculate that rough edges of silica particles due to micro-abrasion may damage the epithelial lining and promote pathogen colonization. Together with the high susceptibility for viral infections, which often followed by bacterial pneumonia, this may explain an increase in acute lower respiratory infections of bacterial origin in young children.

We believe that the observed effect most likely reflects the increase in severity of acute respiratory infections and exacerbation of preexisting asthma-related conditions. In the absence of surveillance systems that can continuously and efficiently monitor the health effects associated with various environmental and socio-economic factors, we were using health outcomes that often reflect the most severe conditions, which require urgent ER visits and hospitalizations. We also anticipate that young children with pneumonia and asthma-related conditions are more likely to be hospitalized than older children or children with acute upper respiratory infection. In our recently completed four-year longitudinal study of Quito's children aged from 6 to 36 months, we recorded 4,450 cases of AURI and 518 physician-diagnosed cases of pneumonia, out which six (1.2%) cases required hospitalization (Sempertegui et al, in preparation). Furthermore, of all the AURI episodes, 102 (2.3%) progressed into ALRI. Therefore, we anticipate that for one episode of hospitalization due to pneumonia there are approximately 86 cases of mild pneumonia. This observation helped us to assert the severity of respiratory conditions. In fact the observed effect was very similar with reported increase in AURI (2.6 times), ALRI (2.5 times) and asthma (2.1 times) eruptions of Tungurahua in 1999 [37].

The observed increase in respiratory infections in April of 2000 was most likely triggered by aerosols and ash falls produced by Pichincha eruptions. However, we recognize that the main limitation of our analysis is the absence of direct measures of exposure. It is also plausible that ash particles deposited during 1999 ash falls can be re-suspended in the air without elevated volcanic activity. Unfortunately, in Quito, systematic monitoring of air quality for critical air pollution was initiated after 2000, so we can only rely on indirect proxies such as satellite imagery records for ash emissions and daily records on seismic activity of Pichincha to establish the timing of high exposure [21]. We assessed the relations between ER visits and timing of high exposure in a manner of ecological analysis, limitations of which pose caution on our conclusions. First of all, health effects of volcanic aerosols and ambient traffic-related pollution are difficult to disentangle. Secondly, volcanic activity is a continuous process, in which the delayed health effects of one event may overlap with others. Thirdly, a seasonal elevation in incidence of respiratory infections and pneumonia could coincide with the eruption and therefore bias the results. Finally, temporal variations in ER visits could be prompted by a number of social factors (e.g. weekends, holidays, strikes) as well as by changes in perception associated with volcano alerts. Although potential confounding by air pollution, continuous volcanic activity, seasonal infection rates and reporting bias could have affected our study, we believe that their role is not substantial.

Volcanic ashes worsened the already poor air quality in Quito [14]. Typically, the chemical composition of ashes could be distinguished from by-products of man-made air pollutants in urban settings. However, one of the most predominant and routinely measured components, particular matter of 10 micron in diameter and larger (PM_10_), are in both volcanic ashes and traffic-related products of incomplete combustion. In fact, a few samples of PM_10 _concentrations collected in urban Quito in the late-fall of 1999 demonstrated an extremely high level of PM_10 _in both the northern and southern parts of the city. For instance, during the 1999 Pichincha eruption PM_10 _increased progressively from 58 μg/m^3 ^in September 30, to 407 μg/m^3 ^in October 6–7, to 1487 μg/m^3 ^in November 26, 1999, exceeding the allowed level by 8 to 28 times [25]. Unfortunately, specific information of ash composition, transport, and deposition is limited, especially for eruptions prior to 2000 [39]. It has been reported that the ash contained substantial respirable material and an elevated cristobalite content [26,40]. An elementary description of Guagua Pichincha ash given by Baxter [26] for three consecutive eruptions in November 1999, July 2000, and February 2002, is based on percentage of PM_10 _in ash that ranged from 7.9 to 13.6, not too dissimilar to Mount St Helens 1980 ash composition [5]. The data from two other Ecuadorian eruptions of Tungurahua and El Reventador volcanoes in 2001 suggested that in Quito area the ash layer was mainly composed of medium to fine ash particles with about 20% free crystals [41]. This study also indicates that the ash re-suspended during the windy afternoons caused ocular irritation, respiratory troubles, and other health problems.

**Figure 4:**
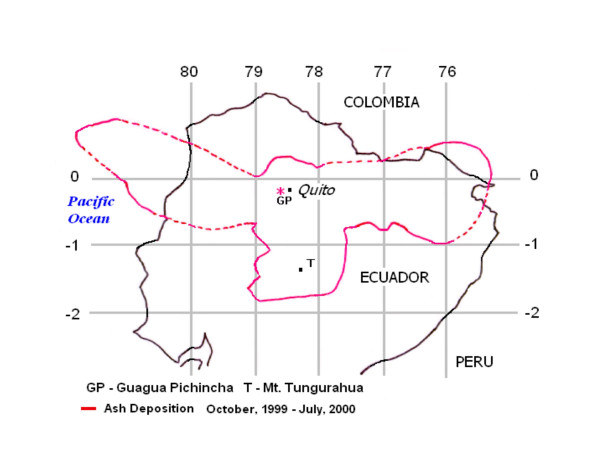
A composite map of ash deposition from Pichincha eruptions (October 1999 – July 2000) demonstrating the extensive area affected by ashfalls. Footnotes: The map has been modified from the images of the Volcano Ash Advisory Archive [21], courtesy of Dr. Yepes, the Geophysical Institute of Ecuador.

Quito was affected by several episodes of ash emission in October – December of 1999, April 2000, and July 2000 (see Figure 4). It is possible that our estimates of the ER rates before and after the eruptions in April could have been elevated by these prior eruptions, and then we probably underestimated the effect. It is also plausible, that we were able to detect the effect of volcanic activity due to little or no rain, which would have cleansed the air during the days of eruption (see Figure 1). Again, additional studies are needed to better quantify the effect of ash fallouts and related pollutants.

The seasonal variations in respiratory infections in Ecuador, and specifically in Quito, are poorly documented. In our own research, we noticed that the incidence of acute upper respiratory infections has no pronounced seasonal variations. The incidence of pneumonia however had a defined seasonal peak in December, January, and February with the lowest level in May, June, and July (Naumova, unpublished data). We believe that an increase of ER visits observed in late April and early May is unlikely to be obscured by typical seasonal variations in the incidence of respiratory infections.

This study was conducted in a government-subsidized hospital, easily accessible by public transportation, providing free care exclusively for children. Although, the quality of medical care utilization may depend on socio-economic status, the main routines of ER visitations are fairly standard in city hospitals. The change in attitudes and perceptions of potential risks associated with volcano eruption in patients, their parents, and physicians could potentially affect the pattern of ER visitations [38]. However, little is known about behavioral aspects of medical service utilization in Quito. Important studies performed by the University of South Florida suggest that attitudes of high resilience, e.g. the ability to withstand stresses are quite dominant in small Ecuadorian towns affected by volcanic activity of Tungurahua and Chimborazo [10]. Furthermore, before the April eruptions, a series of yellow alerts (which assumed to be prepared to close schools and businesses and to evacuate when necessary, to know the plan of alternative and evacuation routes, locations of shelters and hospitals) [11] could have warned both physicians and families disrupted by the disaster, and therefore over-utilization is somewhat implausible. Strikes of medical personnel or closing of hospitals (events that could artificially lower the rate of ER visits) can occur in Quito, however the days with extremely low counts are not systematic and no records of strikes in Baca Ortiz or any city hospitals before or after the April eruption of 2000 are known. We believe that the observed increase in ER visits at the Baca Ortiz Hospital after the volcano eruption reflect a plausible health effect on children from families with low and moderate income living in all communities of Metropolitan Quito.

## Conclusion

We documented significantly increased levels of respiratory disease in Quito children after a volcanic eruption using hospital records. Sound environmental decisions require estimates of the burden of disease or other externalities suffered by the population. In Ecuador, continuous monitoring of air quality is in its infancy, population based surveillance data relevant to environmentally linked diseases are scanty, and often not usable except for descriptive purposes. The quality of passively reported information obtained by the authorities is variable. Quito hospitals do not collect data from emergency room visits or hospitalizations in a manner suitable for detailed analysis electronically and records abstraction was performed manually. Nevertheless, the fraction of missing data in the compiled database is ~5%). It is feasible to prospectively collect highly specific information in ER of Baca Ortiz Hospital in electronic format and at low cost. The next natural steps of this study are to abstract historical ER records that will cover a longer time span including dramatic volcanic eruptions of 1999, the period of volcano dormancy and initiate prospective data collection with the emphasis on linking health and environmental information. We believe that at this stage remote sensing data can provide highly valuable information regarding the characterization of ash deposition [42]. However, remotely sensed data on ash transport and deposition might have some limitations. For example, current techniques are not uniformly effective in classifying volcanic ash and may falsely interpret meteorological clouds as volcanic ash clouds and conversely [43]. Future interdisciplinary studies are required to convert remote sensing data and other data related to volcanic activity into information relevant to public health needs. The efforts to prospectively acquire human health and disease information from sentinel populations, or from representative populations, will build disease surveillance capacity which will greatly improve our knowledge and decision making at many levels.

## Abbreviations

particular matter (PM), emergency room (ER), acute upper respiratory infection (AURI), acute lower respiratory infection (ALRI), relative risks (RR), confidence intervals (CI), lower boundary of confidence interval (LCI), upper boundary of confidence interval (UCI), tumor necrosis factor (TNF)

## Competing interests

The author(s) declare that they have no competing interests.

## Authors' contributions

EN and BE designed the study and conducted the analysis. JG, FS, and BE organized the study. HY participated in its design and contributed towards characterization of exposure. EJ organizad the data abstraction and revised the manuscript critically. GK and JJ performed data abstraction and data analysis. All authors read and approved the final manuscript.
